# Macrophage Polarization in the Skin Lesion Caused by Neotropical Species of *Leishmania* sp

**DOI:** 10.1155/2021/5596876

**Published:** 2021-04-10

**Authors:** Carmen M. Sandoval Pacheco, Gabriela V. Araujo Flores, Kadir Gonzalez, Claudia M. de Castro Gomes, Luiz F. D. Passero, Thaise Y. Tomokane, Wilfredo Sosa-Ochoa, Concepción Zúniga, Jose Calzada, Azael Saldaña, Carlos E. P. Corbett, Fernando T. Silveira, Marcia D. Laurenti

**Affiliations:** ^1^Departamento de Patologia, Laboratório de Patologia de Moléstias Infecciosas, Faculdade de Medicina, Universidade de São Paulo, Av. Doutor Arnaldo 455, 01246-903, Cerqueira César, São Paulo, SP, Brazil; ^2^Departamento de Parasitología Molecular, Instituto Conmemorativo Gorgas de Estudios de la Salud, Ave. Justo Arosemena, 0816-02593 Calidonia, Panama; ^3^São Paulo State University (UNESP), Institute of Biosciences and Institute for Advanced Studies of Ocean, São Vicente, SP, Brazil; ^4^Instituto de Investigación en Microbiología, Universidad Nacional Autónoma de Honduras, Tegucigalpa, Honduras; ^5^Departamento de Vigilancia de la Salud, Hospital Escuela, Tegucigalpa, Honduras; ^6^Facultad de Medicina Veterinaria, Universidad de Panamá, Campus Harmodio Arias Madrid, Av. Juan Pablo II, Albrook, Panama; ^7^Centro de Investigación y Diagnóstico de Enfermedades Parasitarias, Facultad de Medicina, Universidad de Panamá, Ave. Octavio Méndez Pereira, Panama; ^8^Departamento de Parasitologia, Instituto Evandro Chagas, Belém, PA, Brazil; ^9^Núcleo de Medicina Tropical, Universidade Federal de Pará, Belém, PA, Brazil

## Abstract

Macrophages play important roles in the innate and acquired immune responses against *Leishmania* parasites. Depending on the subset and activation status, macrophages may eliminate intracellular parasites; however, these host cells also can offer a safe environment for *Leishmania* replication. In this sense, the fate of the parasite may be influenced by the phenotype of the infected macrophage, linked to the subtype of classically activated (M1) or alternatively activated (M2) macrophages. In the present study, M1 and M2 macrophage subsets were analyzed by double-staining immunohistochemistry in skin biopsies from patients with American cutaneous leishmaniasis (ACL) caused by *L.* (*L.*) *amazonensis*, *L.* (*V.*) *braziliensis*, *L.* (*V.*) *panamensis* ,and *L.* (*L.*) *infantum chagasi.* High number of M1 macrophages was detected in nonulcerated cutaneous leishmaniasis (NUCL) caused by *L.* (*L.*) *infantum chagasi* (M1 = 112 ± 12, M2 = 43 ± 12 cells/mm^2^). On the other side, high density of M2 macrophages was observed in the skin lesions of patients with anergic diffuse cutaneous leishmaniasis (ADCL) (M1 = 195 ± 25, M2 = 616 ± 114), followed by cases of localized cutaneous leishmaniasis (LCL) caused by *L*. (*L*.) *amazonensis* (M1 = 97 ± 24, M2 = 219 ± 29), *L.* (*V*.) *panamensis* (M1 = 71 ± 14, M2 = 164 ± 14), and *L*. (*V*.) *braziliensis* (M1 = 50 ± 13, M2 = 53 ± 10); however, low density of M2 macrophages was observed in NUCL. The data presented herein show the polarization of macrophages in skin lesions caused by different *Leishmania* species that may be related with the outcome of the disease.

## 1. Introduction

Macrophages have important roles in the immune system and play specific functions related to both innate and acquired immunity. During *Leishmania* infection, macrophages may have a dual role, killing or providing a safe environment for parasites. Thus, these host cells are fundamental in the progress or failure of the infection that relies on the type and magnitude of the host immune response [1–3].

In the vertebrate host, macrophages are found as naïve macrophages (M0), and the microenvironment where these cells survive provides different signals, leading to the development of different macrophage subsets, such as M1 (classically activated macrophages) and M2 macrophages (alternatively activated macrophages) [1, 4, 5]. These both macrophage subsets differ in cytokine production and, consequently, in their functions [6–8]. The microenvironment with IFN-*γ* and TNF-*α* presence may promote M1 subset differentiation, and this macrophage subset is able to present antigen, favoring the production of proinflammatory cytokines and reactive oxygen and nitrogen intermediates; besides, it aids the development of type 1 T helper lymphocytes (Th1). Phenotypically, M1 macrophage subsets express CD68 protein, which is a receptor for oxidized low-density lipoproteins (LDLs). Once CD68 binds to LDLs, M1 macrophages become able to phagocyte the pathogens and produce proinflammatory cytokines. Thus, M1 cells are crucial to eliminate intracellular pathogens, as well as *Leishmania*, by triggering an effective oxidative burst [2, 3, 6, 9–12].

On the other hand, microenvironment with high amounts of Th2 cytokines, such as IL-4, IL-10, and IL-13, favors the development of M2 macrophages. This macrophage subset has a low capacity for presenting antigens, has immunoregulatory properties, and reduces the inflammatory response by suppressing the proliferation and activity of T cells. Comparatively, M2 macrophages have opposed functions to M1 cells, being characterized by low production of IL-12 and high of IL-10, a cytokine associated with the development of adaptive Th2 immune responses. Additionally, M2 macrophages are involved in the remodeling of the extracellular matrix and angiogenesis, promoting tissue repair [2, 7].

Depending on the stimulus, M2 macrophages can be further divided into four subgroups: M2a macrophages that are induced by IL-4 or IL-13 produced mainly by Th2 cells, mast cells and basophils; M2b macrophages induced by immune complexes recognized by Fc receptors as well as agonists of Toll-like receptors (TLR) or IL-1; M2c macrophages induced by IL-10, TGF-*β*, and glucocorticoids that are considered deactivating macrophages; and finally, M2d macrophages induced by TLR agonists through the adenosine A2A receptor, once differentiated induce IL-10 and vascular endothelial growth factors releasing, additionally promote angiogenesis and tumor progression [2, 3, 6, 9–15].

It is well known that different *Leishmania* species trigger different immune responses [16, 17]. Based on the antigenic differences of the *Leishmania* species, a clinical and immunopathological spectrum of American cutaneous leishmaniasis has been described [18, 19]. The most common clinical form is named localized cutaneous leishmaniasis (LCL), which is located in the center of the spectrum. This clinical form can be caused by several species of the subgenus *Leishmania* or *Viannia*, and from the histopathological point of view, this clinical form is characterized by an inflammatory infiltrate formed by lymphocytes, macrophages, and plasma cells with variable parasitism and a mixed cellular immune response. Mucocutaneous leishmaniasis (ML) has been considered the hyperreactive pole of the spectrum, and it is caused by parasites of the subgenus *Viannia*, mainly *L.* (*V.*) *braziliensis* and *L.* (*V.*) *panamensis* and histopathologically is characterized by the presence of lymphocytes with rare parasitism; additionally, a Th1-type cellular immune response with high amounts of proinflammatory cytokines can be identified in such cases. In contrast, the hyporeactive pole of this spectrum is anergic diffuse cutaneous leishmaniasis (ADCL), caused by parasites belonging to the subgenus *Leishmania*, mainly to *L.* (*L.*) *amazonensis* and *L.* (*L.*) *mexicana*, and it can be characterized by a plentiful Th2-type immune response and high amounts of circulating anti-inflammatory cytokines, and histopathologically, it is possible to observe high densities of heavily parasitized macrophages.

In experimental studies, M2 macrophages have been related to the development of pathology, and as a consequence, *L.* (*L.*) *major* and *L.* (*L.*) *amazonensis* survived and multiplied into macrophages [20, 21]. In contrast, M1 macrophages have been related to *in vivo* host resistance in *L.* (*L.*) *mexicana* and *L.* (*V.*) *braziliensis* infection [22, 23]. Therefore, the polarization of macrophages to M1 or M2 subsets is an important factor for the host in the final outcome of the disease; however, to the best of our knowledge, few reports performed a comparative analysis on the impact of macrophage subsets in human cutaneous leishmaniasis caused by different *Leishmania* species. Such study may shed further light on the importance and impact of macrophage subsets during the evolution of human cutaneous leishmaniasis in the American continent.

In this sense, the present study subpopulation of M1 and M2 macrophages were analyzed by double-staining immunohistochemistry in different clinical forms of human American cutaneous leishmaniasis (ACL) caused by *L.* (*L*.) *amazonensis*, *L.* (*V.*) *panamensis*, *L.* (*V.*) *braziliensis*, and *L.* (*L.*) *infantum chagasi*.

## 2. Materials and Methods

### 2.1. Human Skin Biopsies

Thirty-four skin biopsies from patients with ACL, previously diagnosed by clinical, parasitological, and molecular tests [24–26], were collected before treatment. Among them, five biopsies belonged to anergic diffuse cutaneous leishmaniasis (ADCL) caused by *L.* (*L.*) *amazonensis*, four to localized cutaneous leishmaniasis (LCL) caused by *L.* (*L.*) *amazonensis*, ten to LCL caused by *L. (V.) panamensis*, five to LCL caused by *L.* (*V.*) *braziliensis*, and ten to nonulcerated or atypical cutaneous leishmaniasis (NUCL) caused by *L.* (*L.*) *infantum chagasi* (Table 1).

All samples were obtained from the repository of the Laboratory of Pathology of Infectious Diseases, Medical School of University of São Paulo, previously approved by the Ethics of Research Committee of the Medical School, University of São Paulo, Brazil (CAAE:83455317.9.0000.0065, CAAE:12861013.2.0000.0065, CAAE:25714814.0.0000.0065).

### 2.2. *In Situ* Detection of M1 and M2 Macrophages

Double-staining immunohistochemistry reaction was performed to observe M1 and M2 macrophage subsets. Both iNOS (polyclonal, ab15323) and CD68 antibodies (monoclonal, ab955) were used in double-staining immunohistochemistry reaction to identify the M1 subset while IL-10 (polyclonal, ab34843) and CD163 (monoclonal, ab156769) antibodies were used to identify M2 macrophages [2, 7, 11, 27, 28]. All primary antibodies were purchased from ABCAM.

Double-staining immunohistochemistry reaction was performed in two steps. Firstly, histological sections were deparaffinized in xylene for 15 minutes, followed by hydration in a descending series of alcohols. Then, endogenous peroxidase blockade was performed with 3% hydrogen peroxide solution. Antigen retrieval was performed using 10 mMol/L citrate buffer pH 6.0 in a boiling water bath. After these steps, the following primary antibodies, produced in rabbits, added anti-iNOS (1 : 100) and anti-IL-10 (1 : 1500). As a negative control, a solution containing phosphate-buffered saline (PBS) and bovine serum albumin (BSA) with the omission of the primary antibody was used. The slides were incubated in a humidified chamber overnight at 4°C. To develop the reaction, a NOVOLINK™ polymer detection systems kit (RE7280-K, Leica Biosystems, Newcastle, UK) was used. The chromogenic substrate, DAB+H_2_O_2_ (diaminobenzidine with hydrogen peroxide, K3468, DakoCytomation), was added to the tissue, incubated for 5 minutes and briefly counterstained with Harris haematoxylin and immersed in TBS-T (Tris Buffered Saline with 0.05% Tween 20).

In the second step, the following primary antibodies, produced in mice, added anti-CD68 (1 : 400) and anti-CD163 (1 : 200). As a negative control, a solution containing phosphate-buffered saline and bovine serum albumin with the omission of the primary antibody was used. The slides were incubated in a humidified chamber overnight at 4°C; then, HRP mouse polymer (ABCAM, ab210061) was incubated for 30 minutes at room temperature. Slides were incubated with emerald chromogen (ABCAM, ab210061) for 5 minutes at room temperature; then, the slides were rinsed with distilled water, left to dry at room temperature to dehydrate the histological sections, and mounted with limonene mounting medium (ABCAM, ab104141).

#### 2.2.1. Quantitative Morphometric Analysis

Ten sequential fields of each histological section using ×40 objective to give a final magnification of ×400 were photographed in an optical microscope coupled to the computer using the AxioVision 4.8.2 software (Zeiss, San Diego, CA, USA). The immunolabeled cells were quantified considering the pattern of staining as well as cell morphology. Immunostained cells were recorded in the dermal layer of the skin, the area where the inflammatory infiltrate was present. The determination of the cellular density (number of cells per square millimeter) of each marker was determined by the ratio between the immunostained cells and the area of each photo.

#### 2.2.2. Statistical Analysis

Analysis of the data was performed using GraphPad Prism 8.0 software. The Kolmogorov-Smirnov test was employed to assess the normality of samples. *t*-test was used for data with a Gaussian distribution to compare the density of cellular markers. The results were expressed as the mean ± standard error. The one-way ANOVA test was used to compare CD68, CD163 markers, and subpopulations of macrophages (M1 and M2) between the different clinical forms. Differences were considered as statistically significant when *P* < 0.05. Graphics were made using the Origin 9.6.5.169 software.

## 3. Results

In the present study, M1 and M2 subsets of macrophages were analyzed by double-staining immunohistochemistry, being the M1 subset positive for both CD68 and iNOS markers while M2 macrophages positive for both CD163 and IL-10 markers.

**Figure 1:**
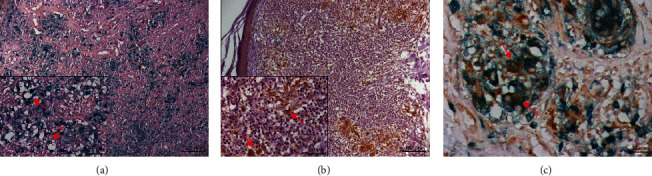
Histological sections of the skin lesion from nonulcerated or atypical cutaneous leishmaniasis (NUCL) processed by double-staining immunohistochemistry showing (a) CD68^+^ cells (blue), (b) iNOS^+^ cells (brown), and (c) M1 macrophages (CD68^+^/iNOS^+^) (×400). The red arrows show immunostained cells for the different markers.

**Figure 2:**
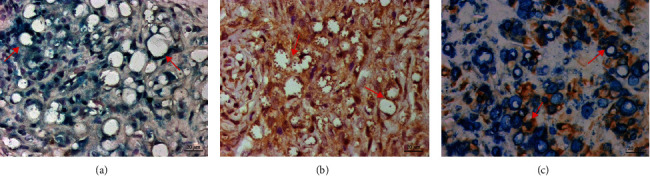
Histological sections of the skin lesion from anergic diffuse cutaneous leishmaniasis (ADCL) processed by double-staining immunohistochemistry showing (a) CD163^+^ cells (blue), (b) IL-10^+^ cells (brown), and (c) M2 macrophages (CD163^+^/IL-10^+^) (×400). The red arrows show immunostained cells for the different markers.

M1 and M2 macrophages were observed in the dermis of the skin lesion from different clinical forms used in this study (Figures 1 and 2).

The quantitative morphometric analysis showed that the density of M1 macrophages was 195 ± 25 cells/mm^2^ for ADCL caused by *L.* (*L.*) *amazonensis*, 97 ± 24 cell/mm^2^ for LCL by *L.* (*L.*) *amazonensis*, 71 ± 14 cell/mm^2^ for LCL by *L.* (*V.*) *panamensis*, 50 ± 13 cell/mm^2^ for LCL by *L.* (*V.*) *braziliensis*, and 112 ± 12 cell/mm^2^ for NUCL by *L.* (*L.*) *infantum chagasi*.

**Figure 3:**
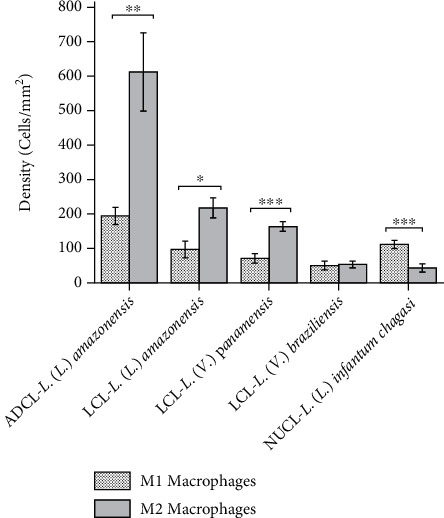
Cellular density (average ± standard error) of M1 and M2 macrophages in the skin lesion of different clinical forms of American cutaneous leishmaniasis, anergic diffuse cutaneous leishmaniasis (ADCL) by *L.* (*L.*) *amazonensis,* localized cutaneous leishmaniasis (LCL) by *L.* (*L.*) *amazonensis*, *L.* (*V.*) *panamensis*, *L.* (*V*.) *braziliensis*, and nonulcerated cutaneous leishmaniasis (NUCL) by *L.* (*L.*) *infantum chagasi*. ^∗^*P* < 0.05; ^∗∗^*P* < 0.01; ^∗∗∗^*P* < 0.001.

On the other hand, the cellular density of M2 macrophages was 616 ± 114 cell/mm^2^ for ADCL caused by *L.* (*L.*) *amazonensis*, 219 ± 29 cell/mm^2^ for LCL by *L.* (*L.*) *amazonensis*, 164 ± 14 cell/mm^2^ for LCL by *L.* (*V.*) *panamensis*, 53 ± 10 cell/mm^2^ for LCL by *L.* (*V.*) *braziliensis*, and 43 ± 12 cell/mm^2^ for NUCL by *L.* (*L.*) *infantum chagasi* (Figure 3).

Comparatively, it was observed that the density of M2 macrophages was higher than M1 in ADCL by *L.* (*L.*) *amazonensis* and also in LCL caused by *L.* (*L.*) *amazonensis* and *L.* (*V.*) *panamensis* (*P* < 0.05). In contrast in NUCL, the density of M1 was higher than that M2 macrophages (*P* < 0.001). The LCL caused by *L.* (*V.*) *braziliensis* showed no statistical difference between M1 and M2 macrophage subsets (*P* > 0.05).

Additionally, the ratio between M1 and M2 macrophages was calculated, and it was observed that the M1:M2 ratio was higher in NUCL (2.605) than in the other clinical forms, ADCL (0.317) and LCL caused by *L.* (*L.*) *amazonensis* (0.443)*, L.* (*V.*) *panamensis* (0.433), and *L.* (*V.*) *braziliensis* (0.943).

**Figure 4:**
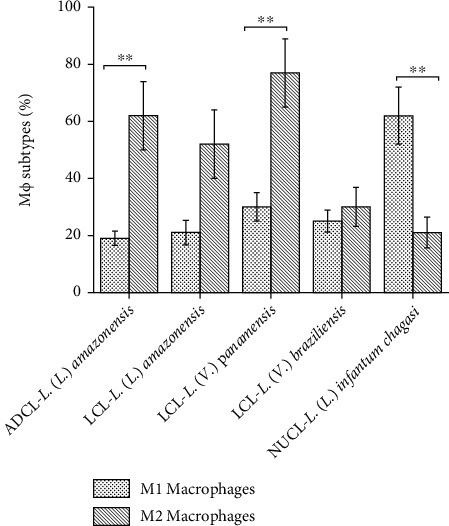
Percentage of M1 and M2 cells inside to total macrophages in the skin lesion of different clinical forms of American cutaneous leishmaniasis, anergic diffuse cutaneous leishmaniasis (ADCL) by *L.* (*L.*) *amazonensis*, localized cutaneous leishmaniasis (LCL) by *L.* (*L.*) *amazonensis*, *L.* (*V.*) *panamensis*, *L.* (*V.*) *braziliensis*, and nonulcerated cutaneous leishmaniasis (NUCL) by *L. (L.) infantum chagasi.*

Considering the percentage of M1 and M2 cells inside to the macrophage population, it is possible to observe that the percentage of M1 cells is lower in ADCL (18%), LCL by *L.* (*L.*) *amazonensis* (21%), and LCL by *L.* (*V.*) *panamensis* (26%) compared to the percentage of M2 cells (57%, 48% and 59%, respectively). On the other side, in NUCL caused by *L.* (*L.*) *infantum chagasi*, the percentage of M1 (53%) is higher than M2 cells (20%) (*P* < 0.01). However, in LCL by *L.* (*V.*) *braziliensis*, the percentage of M1 and M2 cells was similar (26% and 28%, respectively) (Figure 4). Nevertheless, the absence of colocalization of the different markers in double-staining immunohistochemistry reaction, 25% of total macrophages in ADCL by *L.* (*L.*) *amazonensis*, 31% in LCL by *L.* (*L*.) *amazonensis*, 15% in LCL by *L.* (*V.*) *panamensis*, 46% in LCL by *L.* (*V.*) *braziliensis*, and 27% in NUCL by *L.* (*L.*) *infantum chagasi* was not characterized neither as M1 nor M2 cells (Supplementary Table (available here)).

## 4. Discussion

The final outcome of leishmaniasis is multifactorial and depends on the physiology of the host, type of immune response, specie, and virulence of *Leishmania* species. The entry of the parasite into the host cell, the establishment of infection, and the development of the disease involves different steps that may determine the success of the infection, as well as the development of different clinical forms of leishmaniasis [19, 29, 30].

Depending on the interaction between innate cells with T cells, the amount of cytokines produced, and the duration of exposure to parasitic antigens, macrophages can express different functional properties in response to this microenvironment, showing a polarization state that may be related to pathology or self-healing processes [6, 7, 31]. During *Leishmania* infection, macrophage subsets have opposed roles, and M1 macrophage is associated with the elimination of internalized parasites, while M2 is related to the maintenance of the parasite in the intracellular compartment [3]. Thus, in this study, the functional characteristics of macrophages were analyzed *in situ* in different clinical forms of cutaneous leishmaniasis caused by *L*. (*L*.) *amazonensis*, *L*. (*V*.) *panamensis*, *L*. (*V*.) *braziliensis*, and *L*. *(L*.) *infantum chagasi*, and their involvement with the development of different clinical forms was assessed.

Higher density of M2 than M1 macrophages was observed in the skin lesions of patients affected by ADCL caused by *L*. (*L*.) *amazonensis* and LCL caused by *L*. (*L*.) *amazonensis* and *L.* (*V*.) *panamensis*, as observed in Figure 3. Between *L*. (*L*.) *amazonensis* and *L*. (*V*.) *panamensis* infection, a higher amount of M2 macrophages was observed in the ADCL compared to the LCL caused by both species (*P* < 0.05). ADCL is a clinical form caused by *L*. (*L*.) *amazonensis* and *L*. (*L*.) *mexicana* in the New World. It is characterized by a primary lesion, which slowly spreads involving several areas of the skin. The inflammatory infiltrate displays a large number of highly parasitized and vacuolated macrophages, a histopathological characteristic also presented in the LCL caused by *L.* (*L.*) *amazonensis*; however, the intensity of the inflammatory process is lower than in ADCL, additionally in the histological section of the skin of patients with LCL it is possible to observe an inflammatory infiltrate characterized by both plasma cells and T lymphocytes, suggesting a better outcome than ADCL [32]. According to this, the results showed a high number of total macrophages in the ADCL, mainly the M2 subset, regarding the other clinical forms analyzed. In addition, it is possible to note that *L*. (*L*.) *amazonensis* is able to drive the infection from the center of the clinical spectrum that corresponds to the LCL towards the anergic pole of the infection spectrum that corresponds to the form of ADCL, which is one of the most severe clinical forms of leishmaniasis [24, 30]. Silveira et al. showed that ADCL represents the pole of cellular hyposensitivity, indicating that affected patients display cell-mediated immune responses incapable of controlling *Leishmania* spreading. Besides, ADCL patients have preferential activation of a Th2-type immune response resulting in the production of anti-inflammatory cytokines such as IL-4 and IL-10 [18, 19] which can be correlated with a large number M2 macrophages described in the present study. Similar results, high expression of M2 macrophages, were also observed in the anergic pole of leprosy, a chronic disease caused by *Mycobacterium leprae* that is characterized by the presence of vacuolated cells with variable amount of bacillus and development of Th2 immune response that stimulates a suppressive immune response [33].

On the other hand, patients with LCL caused by *L*. (*V*.) *panamensis* present ulcerated lesions and assembled a mixed cellular immune response, with the production of pro- and anti-inflammatory cytokines that is related to the pathology observed in this clinical form of the disease [34]. The inflammatory infiltrate in the LCL caused by this parasite is characterized by the presence of lymphocytes, macrophages, and plasma cells that have a correlation with a moderated size of the skin lesions and parasite density [35, 36]. Thus, in this study, a lower percentage of total macrophages was observed in LCL by *L.* (*V.*) *panamensis* than LCL by *L.* (*L*.) *amazonensis*. However, the results point to the predominance of M2 similar to that observed in the LCL caused by *L*. (*L*.) *amazonensis*, suggesting that even with the presence of M1 macrophages, they are not enough to restrain parasite spreading.

The microenvironment in which macrophages are found provides different signals that activate them, leading to the development of functionally distinct macrophages. Therefore, the presence of Th2 lymphocytes producing IL-4, IL-10, and IL-13 cytokines in *L.* (*L.*) *amazonensis* and *L.* (*V.*) *panamensis* infections may stimulate the polarization macrophages toward the M2 subset, via activation of the enzyme arginase and production of urea and L-ornithine, favoring growth and survival of *Leishmania* in the macrophages and disease progression [1, 3, 37, 38]. The polarization of M2 macrophages in *Leishmania* infection can also be influenced by the parasite species [17]. Farrow et al. in an *in vitro* experimental study using *L*. (*L*.) *amazonensis* and *L*. (*L*.) *major* demonstrated that only M2 macrophages allow parasite growth. Besides, they showed that lipophosphoglycan (LPG) and gp63 from *Leishmania* surface act on M2 macrophages and suppresses the ncRNA genes leaving these cells permissive to infection [20]. In this sense, Lee et al. showed that the failure to cure the cutaneous lesion by *L*. (*L*.) *major* is related to an efficient interaction with M2 macrophages that facilitate the phagocytosis of the parasite suggesting that the preferential infection of this cell type plays a crucial role in the pathogenesis of the disease [21].

Regarding the LCL caused by *L*. (*V*.) *braziliensis*, our results did not show a statistical difference between M1 and M2 macrophages (*P* > 0.05). Despite the small number of macrophages present in these lesions, a similar number of M1 and M2 macrophages were observed. This finding can be associated to the characteristics of the inflammatory infiltrate presents in the lesion caused by *L. (V.) braziliensis* that is formed mainly by T lymphocytes and plasma cells, with a rare number of macrophages and scarce parasitism [18, 19]. Besides, patients have preferential activation of Th1 immune response, probably driven by parasite antigens [17], which is an important factor for controlling infection [39–43].

In NUCL caused by *L.* (*L.*) *infantum chagasi*, it was possible to identify a high density of M1 macrophages over M2, which could be associated to an efficient cellular immune response in the skin of patients. Such patients developed a robust response of CD8^+^ T lymphocytes and IFN-*γ*^+^ cells [26, 44–46] that may control parasite spreading and polarize macrophages to a M1 pole. M1 macrophages have the ability to present antigens and produce and secrete proinflammatory cytokines; furthermore, these activated macrophages can kill *Leishmania* through toxic intermediates of nitrogen and oxygen. Remarkably, the M1 subset inhibits IL-10 production, favoring the development of Th1 lymphocyte response; thus, they are crucial for the elimination of *Leishmania* [2, 3, 6, 9–12, 38].

In post-Kala-azar Dermal Leishmaniasis (PKDL) caused by *L.* (*L.*) *donovani*, it was demonstrated that monocytes decreased the expression of TLR-2 and 4, as well as the production of reactive oxygen and nitrogen species. In addition, monocytes and intralesional macrophages presented high expression of CD206, arginase-1, and PPAR*γ* mRNA, suggesting that macrophage polarization follows to the M2 subtype, sustaining the chronicity in PKDL. However, after therapy, an immunological shift is observed, where macrophages display a profile of the M1 subset, suggesting that repolarization could be considered as a therapeutic approach [47].

Considering the percentage of M1 macrophages regarding the total of macrophage cells, a higher percentage of M1 cells in NUCL caused by *L*. (*L*.) *infantum chagasi* and a lower percentage of M1 cells in ADCL by *L*. (*L*.) *amazonensis* and LCL caused by *L*. (*L*.) *amazonensis* and *L*. (*V*.) *panamensis*. Interestingly, there were a considerable number of macrophages that was not characterized as M1 or M2 macrophages (Supplementary Table), suggesting that different subpopulations of macrophages could be related to the different clinical forms caused by different species of parasite. In this sense, depending on the stimulus, different subtypes of M2 macrophages can be observed. M2a subset can be induced by IL-4 or IL-13, M2b by immune complexes and IL-1, M2c by IL-10 and TGF-*β*, and M2d by TLR agonists [13, 20, 21, 38, 48–52]. Similarly, M1 macrophage subsets can also be categorized in M1a that is considered the classically activated macrophages and M1b the innate-activated macrophages, that is unable to fully develop into an M1a profile, failing to produce IL-12, an essential cytokine for triggering Th1 response [2].

Additionally, the M1:M2 ratio was lower than one in the skin lesions of ADCL patients, followed by LCL caused by *L*. (*L*.) *amazonensis* and *L*. (*V*.) *panamensis*, evidencing the preferential involvement of M2 macrophages in the more severe clinical form of infection (ADCL). However, the M1:M2 ratio in the skin lesions in NUCL caused by *L*. (*L*.) *infantum chagasi* was higher than one showing preferential involvement of M1 macrophages in the most benign clinical form of the disease. On the other hand, the ratio between M1 and M2 cells in LCL by *L*. (*V*.) *braziliensis* was close to one indicating a similar participation of these both cellular types in this skin lesion. These results correlate with the histopathological characteristics that may be responsible for differences in tissue parasitism and *in situ* cellular immune responses triggered by different species of parasites [18, 19, 26, 34].

## 5. Conclusions

Taking together, the results showed a polarization of the M1 and M2 macrophages according to the clinical forms of American cutaneous leishmaniasis with a predominance of M2 macrophages in the most severe clinical form, anergic diffuse cutaneous leishmaniasis caused by *L. (L.) amazonensis*, and a predominance of M1 macrophages in the most benign clinical form of disease, nonulcerated cutaneous leishmaniasis caused by *L. (L.) infantum chagasi*. The importance of the parasite species in the polarization of macrophage subpopulations must be considered.

## Figures and Tables

**Table 1:** Characteristics of the samples used in the present study.

Clinical forms	*Leishmania* specie	*N*° of biopsy	Type of lesion	Endemic area
Anergic diffuse cutaneous leishmaniasis	*L.* (*L.*) *amazonensis*	5	Infiltrative/nodular	Brazil
Localized cutaneous leishmaniasis	*L.* (*L.*) *amazonensis*	4	Ulcerated	Brazil
Localized cutaneous leishmaniasis	*L.* (*V.*) *panamensis*	10	Ulcerated	Panamá
Localized cutaneous leishmaniasis	*L.* (*V.*) *braziliensis*	5	Ulcerated	Brazil
Nonulcerated or atypical cutaneous leishmaniasis	*L.* (*L.*) *infantum chagasi*	10	Nonulcerated	Honduras

## Data Availability

The data used to support the findings of this study are available from the corresponding author upon request.

## Supplementary Materials

Supplementary Materials Supplementary 1: table–density of M1 and M2 macrophage subsets considering the total of macrophages and percentage of M1, M2, and non-M1/M2 macrophages in cutaneous leishmaniasis caused by different species of *Leishmania* parasites.

## References

[1] Arango Duque G., Descoteaux A. (2014). Macrophage cytokines: involvement in immunity and infectious diseases. *Frontiers in Immunology*.

[2] Martinez F. O., Sica A., Mantovani A., Locati M. (2008). Macrophage activation and polarization. *Frontiers in Bioscience*.

[3] Tomiotto-Pellissier F., Bortoleti B. T. . S., Assolini J. P. (2018). Macrophage polarization in Leishmaniasis: broadening horizons. *Frontiers in Immunology*.

[4] Adams D. O. (1989). Molecular interactions in macrophage activation. *Immunology Today*.

[5] Anderson C. F., Mosser D. M. (2002). A novel phenotype for an activated macrophage: the type 2 activated macrophage. *Journal of Leukocyte Biology*.

[6] Lawrence T., Natoli G. (2011). Transcriptional regulation of macrophage polarization: enabling diversity with identity. *Nature Reviews. Immunology*.

[7] Mosser D. M. (2003). The many faces of macrophage activation. *Journal of Leukocyte Biology*.

[8] Murray P. J., Allen J. E., Biswas S. K. (2014). Macrophage activation and polarization: nomenclature and experimental guidelines. *Immunity*.

[9] Guruvayoorappan C. (2008). Tumor versus tumor-associated macrophages: how hot is the link?. *Integrative Cancer Therapies*.

[10] MANTOVANI A., SICA A., SOZZANI S., ALLAVENA P., VECCHI A., LOCATI M. (2004). The chemokine system in diverse forms of macrophage activation and polarization. *Trends in Immunology*.

[11] Tidball J. G., Villalta S. A. (2010). Regulatory interactions between muscle and the immune system during muscle regeneration. *American Journal of Physiology. Regulatory, Integrative and Comparative Physiology*.

[12] Zanluqui N. G., Wowk P. F., Pinge Filho P. (2015). Macrophage polarization in Chagas disease. *Journal of Clinical and Cellular Immunology*.

[13] Chistiakov D. A., Bobryshev Y. V., Nikiforov N. G., Elizova N. V., Sobenin I. A., Orekhov A. N. (2015). RETRACTED: Macrophage phenotypic plasticity in atherosclerosis: the associated features and the peculiarities of the expression of inflammatory genes. *International Journal of Cardiology*.

[14] Wang Q., Ni H., Lan L., Wei X., Xiang R., Wang Y. (2010). Fra-1 protooncogene regulates IL-6 expression in macrophages and promotes the generation of M2d macrophages. *Cell Research*.

[15] Yao Y., Xu X. H., Jin L. (2019). Macrophage polarization in physiological and pathological pregnancy. *Frontiers in Immunology*.

[16] Mendonça S. C. F. (2016). Differences in immune responses against *Leishmania* induced by infection and by immunization with killed parasite antigen: implications for vaccine discovery. *Parasites and Vectors*.

[17] Silveira F. T. (2019). What makes mucosal and anergic diffuse cutaneous leishmaniases so clinically and immunopathogically different? A review in Brazil. *Transactions of the Royal Society of Tropical Medicine and Hygiene*.

[18] Silveira F. T., Lainson R., Corbett C. E. (2004). Clinical and immunopathological spectrum of American cutaneous leishmaniasis with special reference to the disease in Amazonian Brazil: a review. *Memórias do Instituto Oswaldo Cruz*.

[19] Silveira F. T., Lainson R., de Castro Gomes C. M., Laurenti M. D., Corbett C. E. P. (2009). Immunopathogenic competences of *Leishmania* (*V*.) *braziliensis* and *L*. (*L*.) *amazonensis* in American cutaneous leishmaniasis. *Parasite Immunology*.

[20] Farrow A. L., Rana T., Mittal M. K., Misra S., Chaudhuri G. (2011). *Leishmania*-induced repression of selected non-coding RNA genes containing B-box element at their promoters in alternatively polarized M2 macrophages. *Molecular and Cellular Biochemistry*.

[21] Lee S. H., Charmoy M., Romano A. (2018). Mannose receptor high, M2 dermal macrophages mediate nonhealing *Leishmania major* infection in a Th1 immune environment. *The Journal of Experimental Medicine*.

[22] Díaz-Gandarilla J. A., Osorio-Trujillo C., Hernández-Ramírez V. I., Talamás-Rohana P. (2013). PPAR activation induces M1 macrophage polarization via cPLA2-COX-2 inhibition, activating ROS production against *Leishmania mexicana*. *BioMed Research International*.

[23] Vellozo N. S., Pereira-Marques S. T., Cabral-Piccin M. P. (2017). All-trans retinoic acid promotes an M1- to M2-phenotype shift and inhibits macrophage-mediated immunity to *Leishmania major*. *Frontiers in Immunology*.

[24] Campos M. B., Lima L. V. . R., de Lima A. C. S. (2018). Toll-like receptors 2, 4, and 9 expressions over the entire clinical and immunopathological spectrum of American cutaneous leishmaniasis due to *Leishmania* (*V*.) *braziliensis* and *Leishmania* (*L*.) *amazonensis*. *PLoS One*.

[25] Gonzalez K., Calzada J. E., Díaz R. (2019). Performance of immunohistochemistry as a useful tool for the diagnosis of cutaneous leishmaniasis in Panama, Central America. *Parasitology International*.

[26] Sandoval Pacheco C. M., Araujo Flores G. V., Favero Ferreira A. (2018). Histopathological features of skin lesions in patients affected by non-ulcerated or atypical cutaneous leishmaniasis in Honduras, Central America. *International Journal of Experimental Pathology*.

[27] Sica A., Mantovani A. (2012). Macrophage plasticity and polarization: *in vivo* veritas. *The Journal of Clinical Investigation*.

[28] Sulahian T. H., Högger P., Wahner A. E. (2000). Human monocytes express CD163, which is upregulated by il-10 and identical to p155. *Cytokine*.

[29] Carvalho L. P., Passos S., Schriefer A., Carvalho E. M. (2012). Protective and pathologic immune responses in human tegumentary leishmaniasis. *Frontiers in Immunology*.

[30] Silveira F. T., Muller S. R., de Souza A. A. A. (2008). Revisão sobre a patogenia da leishmaniose tegumentar americana na Amazônia, com ênfase à doença causada por *Leishmania* (*V*.) *braziliensis* e *Leishmania* (*L.*) *amazonensis*. *Revista Paraense de Medicina*.

[31] Atri C., Guerfali F. Z., Laouini D. (2018). Role of human macrophage polarization in inflammation during infectious diseases. *International Journal of Molecular Sciences*.

[32] Silveira F. T., Lainson R., Shaw J. J., De Souza A. A., Ishikawa E. A., Braga R. R. (1991). Cutaneous leishmaniasis due to *Leishmania* (*Leishmania*) *amazonensis* in Amazonian Brazil, and the significance of a negative Montenegro skin-test in human infections. *Transactions of the Royal Society of Tropical Medicine and Hygiene*.

[33] de Sousa J. R., de Sousa R. P. M., de Souza Aarão T. L. (2016). *In situ* expression of M2 macrophage subpopulation in leprosy skin lesions. *Acta Tropica*.

[34] Gonzalez K., Calzada J. E., Tomokane T. Y. (2020). *In situ* study of cellular immune response in human cutaneous lesions caused by *Leishmania (Viannia) panamensis* in Panama. *Parasite Immunology*.

[35] González K., Diaz R., Ferreira A. F. (2018). Histopathological characteristics of cutaneous lesions caused by *Leishmania Viannia panamensis* in Panama. *Revista do Instituto de Medicina Tropical de São Paulo*.

[36] Restrepo R., Caceres-Dittmar G., Tapia F. J., Isaza D. M., Restrepo M. (1996). Immunocytochemical and histopathologic characterization of lesions from patients with localized cutaneous leishmaniasis caused by *Leishmania panamensis*. *The American Journal of Tropical Medicine and Hygiene*.

[37] Muxel S. M., Aoki J. I., Fernandes J. C. R. (2018). Arginine and polyamines fate in *Leishmania* infection. *Frontiers in Microbiology*.

[38] Wang L., Zhang S., Wu H., Rong X., Guo J. (2019). M2b macrophage polarization and its roles in diseases. *Journal of Leukocyte Biology*.

[39] Bacellar O., Lessa H.´., Schriefer A. (2002). Up-regulation of Th1-type responses in mucosal leishmaniasis patients. *Infection and Immunity*.

[40] Cardoso T. M., Machado Á., Costa D. L. (2015). Protective and pathological functions of CD8+ T cells in *Leishmania braziliensis* infection. *Infection and Immunity*.

[41] Carvalho L. P., Passos S., Bacellar O. (2007). Differential immune regulation of activated T cells between cutaneous and mucosal leishmaniasis as a model for pathogenesis. *Parasite Immunology*.

[42] de Oliveira C. I., Brodskyn C. I. (2012). The immunobiology of *Leishmania braziliensis* infection. *Frontiers in Immunology*.

[43] Follador I., Araújo C., Bacellar O. (2002). Epidemiologic and immunologic findings for the subclinical form of *Leishmania braziliensis* infection. *Clinical Infectious Diseases*.

[44] Araujo Flores G. V., Sandoval Pacheco C. M., Tomokane T. Y. (2018). Evaluation of regulatory immune response in skin lesions of patients affected by nonulcerated or atypical cutaneous Leishmaniasis in Honduras, Central America. *Mediators of Inflammation*.

[45] Araujo Flores G. V., Sandoval Pacheco C. M., Sosa Ochoa W. H. (2020). Th17 lymphocytes in atypical cutaneous leishmaniasis caused by *Leishmania* (*L*.) *infantum chagasi* in Central America. *Parasite Immunology*.

[46] Sandoval C., Araujo G., Sosa W. (2021). *In situ* cellular immune response in non-ulcerated skin lesions due to *Leishmania* (*L*.) *infantum chagasi* infection. *Journal of Venomous Animals and Toxins including Tropical Diseases*.

[47] Mukhopadhyay D., Mukherjee S., Roy S. (2015). M2 polarization of monocytes-macrophages is a Hallmark of Indian post Kala-Azar dermal Leishmaniasis. *PLoS Neglected Tropical Diseases*.

[48] Mantovani A., Sozzani S., Locati M., Allavena P., Sica A. (2002). Macrophage polarization: tumor-associated macrophages as a paradigm for polarized M2 mononuclear phagocytes. *Trends in Immunology*.

[49] Wang N., Liang H., Zen K. (2014). Molecular mechanisms that influence the macrophage M1-M2 polarization balance. *Frontiers in Immunology*.

[50] Mills C. D. (2012). M1 and M2 macrophages: oracles of health and disease. *Critical Reviews in Immunology*.

[51] Shapouri-Moghaddam A., Mohammadian S., Vazini H. (2018). Macrophage plasticity, polarization, and function in health and disease. *Journal of Cellular Physiology*.

[52] Zhou D., Huang C., Lin Z. (2014). Macrophage polarization and function with emphasis on the evolving roles of coordinated regulation of cellular signaling pathways. *Cellular Signalling*.

