# Supplementary material for: Macrophage Polarization in the Skin Lesion Caused by Neotropical Species of Leishmania sp
Source: J Immunol Res. 2021 Apr 10;2021:5596876. doi: 10.1155/2021/5596876 (PMC8055412; doi:10.1155/2021/5596876)
Supplement: Supplementary Materials — Supplementary 1: table–density of M1 and M2 macrophage subsets considering the total of macrophages and percentage of M1, M2, and non-M1/M2 macrophages in cutaneous leishmaniasis caused by different species of Leishmania parasites. [file 5596876.f1.docx]

**Supplementary 1. Table –** Density of M1 and M2 macrophage subsets considering the total of macrophages and percentage of M1, M2 and non-M1/M2 macrophages in cutaneous leishmaniasis caused by different species of *Leishmania* parasites.

| **Clinical forms** | **Parasite specie** | **M1** | | **M2** | | **Non-M1/M2** |
| --- | --- | --- | --- | --- | --- | --- |
|  |  | **Density (cells/mm^2^)** | **Percentage (%)** | **Density (cells/mm^2^)** | **Percentage (%)** | **Percentage**  **(%)** |
| Anergic diffuse cutaneous leishmaniasis | *L. (L.) amazonensis* | 195 ± 25 | 18 | 616 ± 114 | 57 | 25 |
| Localized cutaneous leishmaniasis | *L. (L.) amazonensis* | 97 ± 24 | 21 | 219 ± 29 | 48 | 31 |
| Localized cutaneous leishmaniasis | *L. (V.) panamensis* | 71 ± 14 | 26 | 164 ± 14 | 59 | 15 |
| Localized cutaneous leishmaniasis | *L. (V.) braziliensis* | 50 ± 13 | 26 | 53 ± 10 | 28 | 46 |
| Non-ulcerated or atypical cutaneous leishmaniasis | *L. (L.) infantum chagasi* | 112 ± 12 | 53 | 43 ± 12 | 20 | 27 |
